# The Book World of Medicine and Science

**Published:** 1900-03-24

**Authors:** 


### The Book World of Medicine and Science.

A Manual of Chemistry. By A. P. Luff, M.D., B.Sc.Lond., F.R.C.P., and F. J. M. Page, B.Sc.Lond. Illustrated. Revised edition. (London: Cassell and Co., Limited. 1900. Price 7s. 6d. Pp. 541.)

The introduction to the study of organic and inorganic chemistry, which first appeared in 1892, has now been revised and in some parts rewritten, so that its present form considerably differs from that of its first appearance. The object of the book is to put in a concise form "those portions of chemical science that directly or indirectly bear on the study and practice of medicine." The volume is divided into five parts. Part I., being a general introduction to the subject, gives the student in a brief form a very good elementary idea of the theory of chemistry and its relation to physics. Parts II. and III. deal respectively with the non-metals and metals with their principal compounds. Part IV. is devoted to organic chemistry. All this is excellently done, and headings and special points of interest are well indicated by different styles of type. The explanation of such details as electrolysis, preparation of ozone, hardness and softness of water, the halogens, Newlands and Mendelejeef classification, which generally prove a stumbling block to the average student, is treated with a lucidity of expression and a clearness of style that ought not to fail in lightening the labours of the already overladen medical student. We should have thought that such practical teachers as the authors would have learnt the uselessness of referring the student, as he generally exists, to another book for fuller details. Thus, in treating of Dumas' method of finding the vapour density, the reader is referred to "Quantitative Analysis," Clowes and Coleman, page 421. Part V., the last section of the book, is of a practical nature, and deals with chemical problems, analysis of a simple salt, and preparation of typical salts. The only objection to including in one volume both the theoretical and the practical sides of such a subject is that it exposes the book to an undue amount of rough usage and bad wear. We congratulate the authors on having succeeded in their object of producing a thoroughly reliable elementary text-book, which expounds the fundamental principles of chemical science in a manner at once interesting and scientific, and we can recommend this book not only to students, but to such medical men as wish to renew their acquaintance with chemistry, especially those portions treating of the drugs and medicines that they daily use. An excellent and complete index completes the manual.

Mentally-Deficient Children. By G. E. Shuttleworth, B.A., M.D. Second edition. Crown 8vo. 180 pages. (London: H. K. Lewis. 1900.) Price 5s. nett.

The appe rance of a new edition of this little book, which was out of print, shows that there is a continued demand for such a publication. In this edition two chapters have been added. Under the heading of "Defective and Epileptic Children" will be found in Chapter III. a *resumé* of the inquiry of the Departmental Committee appointed by the Education Department in 1896 to inquire into the existing systems for the education of feeble-minded and defective children, not idiots or imbeciles; in Chapter IV. is given the practical scheme for special instruction adopted by several school authorities, especially th› School Board for London. It is hardly necessary to add that this manual will interest not only medical men, but all those who have to train and treat mentally-deficient children. It gives in a concise but clear form a short retrospect of the movement at home and abroad which has led to the recognition of the necessity of a special education for these unfortunate children, and also shows by illustrative examples how much improvement, both mental and physical, may be effected by this means. In the appendix will be found the clauses of the Act, which is the outcome of the recommendations of the Committee above mentioned. The "get-up" of the book leaves nothing to be desired: a full index, both of subjects and names and chapters, makes reference a simple matter.

